# Curse and blessing: upregulation of oncogenes upon treatment with hypomethylating agents in myelodysplastic syndrome

**DOI:** 10.1038/s41392-022-01143-3

**Published:** 2022-08-09

**Authors:** Juliane Grimm, Mascha Binder

**Affiliations:** Department of Internal Medicine IV, Oncology/Hematology, University Hospital Halle (Saale), Halle (Saale), Germany

**Keywords:** Haematological cancer, Epigenetics

In a recently published study in *The New England Journal of Medicine*, Liu and colleagues were able to demonstrate that—apart from the anti-leukemic effects previously reported for hypomethylating agents (HMAs)—treatment with these drugs can also lead to demethylation and subsequent upregulation of oncogenes resulting in shorter survival in patients with myelodysplastic syndrome (MDS).^1^

The HMAs azacitidine and decitabine have become established treatment options for patients with MDS and acute myeloid leukemia (AML) over the last almost 20 years. Originally, these agents were developed as classical cytostatic drugs. However, it soon became evident that these chemicals potently contribute to DNA demethylation. Although their molecular mechanism of action has not been fully understood until today, it is broadly accepted that the genome-wide demethylation leads to the reactivation of epigenetically silenced tumor suppressor genes and subsequent upregulation of their gene products which contributes to the anti-tumor effect of HMAs.^2^ Despite the uncontested global character of DNA demethylation induced by these drugs, simultaneous deregulation of oncogenes has not been shown previously.

Liu et al. investigated the impact of HMA treatment on the transcription factor SALL4 in MDS patients. SALL4 has some important prerequisites making it a promising target to study in the context of MDS and HMA treatment. First of all, *SALL4* is aberrantly expressed in different myeloid neoplasms.^1,3^ Functionally, it appears to activate the Wnt/β-catenin signaling pathway which resulted in MDS-like phenotypes in a murine model of *SALL4* overexpression.^3^ Perhaps most importantly, its expression has been shown to depend on the methylation status of the gene.^4^ Analyzing two cohorts of newly diagnosed MDS patients with matched bone marrow samples before treatment initiation and after three to five cycles of HMA therapy, Liu et al. demonstrated that *SALL4* expression was already elevated prior to treatment initiation compared to healthy controls. The pre-treatment *SALL4* expression levels, however, were not informative in terms of outcome prediction. In 30 to 40% of the investigated MDS cases, upregulation of *SALL4* mRNA levels was detected after treatment with HMAs. Although no correlation between *SALL4* expression dynamics and clinical response was evident, patients with treatment-induced upregulation of *SALL4* experienced significantly shorter overall survival in both MDS cohorts.

To further characterize the functional link between global DNA hypomethylation resulting from azacitidine or decitabine treatment and *SALL4* upregulation, the authors explored the methylation status of the gene. Previously, it had been demonstrated that the methylation of the CpG island—located between the 5′ untranslated region (UTR) and the first intron of *SALL4*—inversely correlated with its expression.^4^ To identify the exact CpG region which mediates the effect on *SALL4* expression, Liu et al. applied a novel technique called CRISPR-DiR. This method was developed based on the observation that specific non-coding RNAs are able to interact with the de novo DNA methyltransferase 1 (DNMT1) and consequently inhibit its enzymatic activity. The necessary structural features were introduced into guide RNAs enabling them to recruit an enzymatically inactived Cas9 protein and simultaneously bind and block the DNMT1 activity at a specific targeted site. This site-specific inhibition of de novo DNA methylation results in DNA hypomethylation at the desired region after cell division.

Using CRISPR-DiR in an AML and a hepatocellular carcinoma cell line, the authors were able to identify a specific region in the 5´-UTR CpG island that—upon demethylation—upregulated *SALL4*. Interestingly, treatment of these cell lines with decitabine led to comparable demethylation at this CpG island and a dose-dependent increase of *SALL4* expression. This indicated that HMA-mediated demethylation represents a potential mechanism of deregulation of this oncogene. Consequently, Liu et al. investigated the *SALL4* methylation status in MDS patients treated with HMAs demonstrating reduced methylation at the relevant 5′-UTR CpG site in patients with elevated *SALL4* expression after treatment with azacitidine or decitabine.

**Fig. 1:**
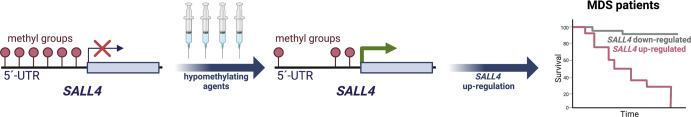
Schematic of the findings reported by Liu et al. Briefly, the study demonstrates that treatment of myelodysplastic syndrome (MDS) patients with hypomethylating agents leads to demethylation of a CpG site in the 5′ untranslated region (UTR) of the oncogene *SALL4* resulting in increased *SALL4* expression which correlates with impaired prognosis in these patients. The figure was created with BioRender.com

Liu et al. establish *SALL4* as a model oncogene upregulated as a consequence of the genome-wide hypomethylation mediated by HMA treatment (Fig. 1). The de-repression of oncogenes in response to HMAs might limit their anti-tumor effects and lead to adverse outcomes, however, it could also be understood as starting point for the establishment of novel therapeutic approaches targeting these oncogenes in MDS patients that do not respond to treatment with azacitidine or decitabine.

Clearly, more work needs to be done to better understand the impact of HMA treatment on the expression levels of oncogenes, since not all MDS patients examined by Liu et al. did demonstrate *SALL4* upregulation in response to HMA therapy. Apart from the complex epigenetic mechanisms behind the anti-tumor effects of HMAs, additional facets of the wide area of action of these drugs seem to be indispensable for the success or failure of hypomethylating therapy in myeloid neoplasms. Our group and others have previously demonstrated that HMAs possess potent immunomodulatory functions which could mediate disease control in patients treated with azacitidine.^5^

The study of Liu et al. makes an important step towards a better understanding of the molecular mechanism underlying HMA treatment. It could also pave the way for novel therapeutic concepts tackling resistance to these drugs. Further investigations have to demonstrate whether HMA mediated DNA demethylation and subsequent de-repression of oncogenes may be a universal mechanism which can be recapitulated in the majority of MDS and AML patients, and which targets may be most affected. The latter may inspire innovative drug combinations ultimately improving the outcome of patients with MDS and AML.
